# Diaphragmatic Reconstruction with Strattice^TM^ Biologic Mesh After Thoracic Oncologic Resection: A 10-Year Single-Center Retrospective Cohort Study

**DOI:** 10.3390/life16101597

**Published:** 2026-09-24

**Authors:** Ivana Vasic, Yolana L. E. Moonsamy, Alexis S. King, Pranita Mishra, Michael T. Ou, Elaine Liang, Jeffrey B. Velotta

**Affiliations:** 1 Department of Surgery, University of California San Francisco East Bay, Oakland, CA 94602, USA; yolanalana.l.moonsamy@kp.org; 2 Division of Thoracic Surgery, Kaiser Permanente Oakland Medical Center, Oakland, CA 94611, USA; jeffrey.b.velotta@kp.org; 3 Division of Research, Kaiser Permanente, Oakland, CA 94588, USA; alexis.x1.thomas@kp.org (A.S.K.); pranita.mishra@kp.org (P.M.); michael.x.ou@kp.org (M.T.O.); 4 Department of Surgery, University of California San Francisco, San Francisco, CA 94602, USA; 5 Kaiser Permanente Bernard J. Tyson School of Medicine, Oakland, CA 91101, USA; elaine.liang@kp.org; 6 Department of Surgery, School of Medicine, University of California San Francisco, San Francisco, CA 94143, USA

**Keywords:** Strattice, diaphragmatic hernia repair, biologic mesh

## Abstract

(1) Background: Large diaphragmatic defects following thoracic oncologic resection often require mesh reconstruction. Data regarding biologic mesh for this application remain limited. We evaluated clinical outcomes and durability of Strattice™, a non-cross-linked porcine acellular dermal matrix, for diaphragmatic reconstruction. (2) Methods: In this single-center, retrospective cohort study without a synthetic mesh comparator, we reviewed health records of 65 adults who underwent diaphragmatic reconstruction with Strattice™ mesh at a high-volume thoracic surgery center between 2014 and 2024. (3) Results: Mean hospital length of stay was 13.2 days. Atrial fibrillation was the most common post-operative complication, occurring in 44.6% of the patients. No diaphragmatic reconstruction recurrence or mesh replacement was observed during the available follow-up. (4) Conclusions: No reconstruction recurrence or mesh replacement was identified during the available follow-up, and wound infection was rare, supporting the feasibility of Strattice™ mesh for diaphragmatic reconstruction in this single-center cohort study. Strattice™ provided durable diaphragmatic reconstruction with a low observed wound infection rate and no identified reconstruction failure, supporting its feasibility for complex thoracic oncologic reconstruction.

## 1. Introduction

Large diaphragmatic defects resulting from oncologic resection pose a significant reconstructive challenge. While smaller defects may be suitable for primary repair, larger full-thickness defects often require reinforcement or patch reconstruction to restore the diaphragm’s integrity, maintain respiratory mechanics, and ensure a durable separation of the thoracic and abdominal cavities. Diaphragmatic reconstruction is particularly important following extensive oncologic resection, as inadequate restoration of this anatomic barrier can result in displacement or herniation of abdominal viscera into the thoracic cavity and impairment of respiratory function. The reconstructive material must provide sufficient mechanical stability while accommodating the stress of continuous motion associated with respiration. Despite the importance of these characteristics, there is currently no clear consensus about the optimal prosthetic material for diaphragmatic reconstruction, and material selection remains largely dependent on surgeon preference and institutional experience [1]. Historically, synthetic materials, most commonly polytetrafluoroethylene (PTFE) or expanded PTFE (GoreTex), have been preferred due to their tensile strength, resistance to deformation, and relative ease of use [1]. However, these materials, while mechanically robust, are not without risk. Reported infection rates for synthetic meshes vary widely from 0.1% to 15%, particularly in contaminated fields, prior chemoradiation, or extensive soft tissue dissection [2]. Complications such as infection, chronic inflammation, erosion, and mesh-related issues can cause serious health problems and sometimes require removal of the mesh. These adverse events can significantly affect oncologic treatment by causing delays or interruption in the subsequent therapies. These concerns have prompted interest in reconstructive materials capable of providing mechanical support while also becoming incorporated into native tissue. This consideration is especially relevant in complex thoracic oncologic resections, in which prosthetic material is often placed in patients who subsequently require additional systemic or local therapy.

Biologic meshes, including acellular dermal matrices (ADMs), offer a potential alternative, with improved biocompatibility and resistance to infection. Strattice™ (LifeCell), a non-cross-linked porcine acellular dermal matrix, has been extensively used in abdominal wall reconstruction, chest wall reconstruction, and complex soft tissue repairs, where it has demonstrated favorable integration, resistance to infection, and long-term durability [3]. In addition, biologic meshes like Strattice™ promote neovascularization and cellular infiltration, potentially leading to more physiologic tissue remodeling and reduced foreign body reaction [1]. Experimental evaluation of porcine acellular dermal matrix has demonstrated cellular infiltration and blood vessel formation within the implanted scaffold, with progressive remodeling and new collagen deposition over time [4]. Histologic evaluation of implanted Strattice™ mesh has demonstrated fibroblast infiltration and revascularization, supporting the biologic basis for incorporation of the matrix into host tissue [5].

Despite these theoretical advantages, biologic mesh is less commonly employed for diaphragmatic reconstruction, and data evaluating its performance in this specific context remain limited. Patients undergoing diaphragmatic resection for thoracic malignancies, particularly pleural mesothelioma, constitute a high-risk surgical population. They often present with substantial comorbidity burden, extensive disease burden, prior chemotherapy or radiation exposure, and large composite resections involving adjacent structures [4,5]. These factors may increase susceptibility to postoperative complications, including pneumonia, prolonged ventilation, atrial fibrillation, and wound infections [6].

Given the growing interest in biologic mesh and the paucity of specific outcomes data in diaphragm reconstruction, we aimed to evaluate the safety, durability, and clinical outcomes associated with Strattice™ mesh for diaphragmatic reconstruction following oncologic resection. Utilizing a 10-year retrospective cohort from a high-volume tertiary referral thoracic surgery practice, we assessed postoperative complications, recurrence, mesh durability, and predictors of clinical outcomes. This study represents one of the largest experiences of biologic mesh in diaphragmatic reconstruction and seeks to clarify the role of Strattice™ as a viable alternative to synthetic prosthetics in complex thoracic oncologic surgery.

## 2. Materials and Methods

### 2.1. Study Design

We conducted a retrospective cohort study of adult patients who underwent diaphragmatic resection with immediate reconstruction using Strattice™ (LifeCell Corporation, Branchburg, NJ, USA), a porcine acellular dermal matrix, at a single, integrated, high-volume thoracic surgery center between 1 January 2014 and 31 December 2024. The institutional review board approved the study (IRB: 2282736-3). Patient consent was waived due to the retrospective nature of the study.

### 2.2. Patient Selection

Eligible patients included adults undergoing surgical resection of thoracic malignancies with direct diaphragmatic invasion requiring partial or full-thickness diaphragm resection followed by reconstruction with biologic mesh. Patients were identified through operative scheduling records, CPT codes specific to diaphragm resection, and chart review of operative logs. Medical records were subsequently reviewed to confirm that diaphragmatic resection and reconstruction with Strattice™ mesh were performed during the index surgery. Cases involving primary repair without mesh or reconstruction using non-biologic materials were excluded. The date of the qualifying operation was considered the index date for subsequent outcome and survival analyses. Patients with higher-stage disease (stage III and IV) were considered for resection following multidisciplinary tumor board discussion, based on overall good performance status and, in selected cases, palliative intent.

### 2.3. Surgical Technique

Diaphragmatic resection was performed as part of oncologic resection for pleural mesothelioma or other thoracic malignancies with diaphragmatic invasion requiring en bloc resection. Resection was performed by the thoracic surgery service, with diaphragmatic reconstruction performed jointly with plastic surgery service when myococutaneous flap was required. The defect was reconstructed using Strattice™ biologic mesh. The mesh was tailored to the size of the defect and trimmed to a football shape (oval), conforming to the native contour of the diaphragm. Mesh was secured as a single-layer repair with interrupted horizontal mattress sutures (#1 Vicryl, GS-25 needle). Anterolaterally, eight sutures were placed through the mesh and around the adjacent rib, from medial to lateral. Posteromedially, the additional sutures of the same material and technique were placed from the mesh into the pericardiodiaphragmatic attachments, and from the paraspinous region to the anterior mediastinum to manage this technically demanding margin. Prior to mesh inset, patient positioning was adjusted to minimize chest wall laxity and ensure appropriate mesh tension in a more physiologic position, with the goal of limiting postoperative paradoxical motion A tension-free repair technique was prioritized to minimize postoperative herniation or recurrence.

### 2.4. Data Collection

Demographic, clinical, oncologic, operative, and postoperative data were obtained through retrospective chart review. Demographic variables included age at the index operation, sex, race, and ethnicity. Clinical characteristics included body mass index (BMI), smoking status, and comorbidities. Comorbidity was quantified using the Elixhauser comorbidity index both with and without malignancy included in the calculation. Socioeconomic traits were assessed using the Neighborhood Deprivation Index (NDI), categorized into quartiles, with higher ones representing greater neighborhood-level deprivation. For regression analysis, NDI was grouped into lower deprivation (quartiles 1–2) and higher deprivation (quartiles 3–4). Oncologic variables included tumor histology, stage, presence of pleural mesothelioma, and receipt of chemotherapy and radiation treatment. For the regression model, tumor stage was grouped as stage 0–1 vs. stage 2–4. Race and ethnicity were collapsed for regression analyses due to the small number of patients within individual racial/ethnic categories. Operative and postoperative data included length of hospital stay and postoperative complications. Follow-up was calculated from the index operation until death or the last available follow-up.

### 2.5. Outcomes

Postoperative outcomes included atrial fibrillation, myocardial infarction, pneumonia, prolonged mechanical ventilation exceeding 48 h, readmission to the hospital, stroke, wound infection, and return to the operating room. Length of hospital stay was calculated from the date of index operation to the date of hospital discharge. Reconstruction-specific outcomes included the need for diaphragmatic mesh replacement and diaphragmatic reconstruction recurrence. Postoperative surveillance included routine cross-sectional imaging (CT chest and abdomen) every six months for the first two years from the index operation and annually thereafter. Reconstruction recurrence was defined as radiologic or clinical identification of diaphragmatic herniation or reconstruction failure during the surveillance time. Mortality and long-term survival was assessed from the index operation to the end of available follow-up or death.

### 2.6. Statistical Analysis

Patient characteristics and clinical outcomes were summarized using descriptive statistics. Continuous variables were reported as mean with standard deviation (SD) and, where appropriate, median with interquartile range (IQR). Categorical variables were summarized as frequencies and percentages.

Multivariable logistic regression was used to evaluate associations between selected patient and clinical characteristics and postoperative binary outcomes. Candidate covariates included age, sex, race/ethnicity, NDI, cancer stage, and receipt of chemotherapy and radiation treatment. Adjusted odds ratios with 95% confidence intervals (CI) were calculated. For categorical variables, cancer stage 0–1 and NDI quartiles 1–2 were used as reference categories. Outcomes with insufficient event numbers, particularly several reconstruction-specific and postoperative outcomes with zero or one occurrence, were excluded from regression analyses due to unreliable estimation.

Linear regression was applied to evaluate relationships between predefined clinical variables and hospital length of stay. Regression coefficients with corresponding 95% CI and *p*-values were reported. All statistical tests were two-sided, with significance defined as a *p*-value < 0.05.

### 2.7. Survival Analyses

Overall survival was defined as the interval from the index operation to death. Patients who remained alive were censored at the end of available follow-up. Survival distributions were estimated using the Kaplan–Meier product limit method.

Kaplan–Meier survival estimates and corresponding 95% confidence intervals were calculated at 1, 3, and 5 years following the index operation. Median survival and its 95% confidence interval were also estimated. Confidence intervals for survival estimates were calculated using log transformation. The number of patients remaining at risk at 1, 3, and 5 years was also reported.

## 3. Results

### 3.1. Patient Characteristics

A total of 65 patients underwent diaphragmatic resection and reconstruction with Strattice™ biologic mesh between 2014 and 2024 (Table 1). The cohort had a mean age of 71.3 ± 11.6 years, and 42 patients (64.6%) were male. The cohort included 37 White patients (56.9%), 16 Hispanic patients (24.6%), 7 Asian patients (10.8%), 4 Black patients (6.2%), and one patient (1.5%) who identified as Native Hawaiian/Pacific Islander. The mean body mass index was 26.9 ± 4.7kg/m^2^ with a median of 27.0 kg/m^2^ (IQR, 24.0–29.0). Thirty-two patients (49.2%) were former smokers, 33 (50.8%) were nonsmokers, and no patient was characterized as a current smoker. The mean Elixhauser comorbidity score excluding cancer was 4.9 ± 2.4, while the mean score including cancer was 6.3 ± 2.5.

Neighborhood deprivation index distribution included 21 patients (32.3%) in the first quartile, 19 (29.2%) in the second quartile, 17 (26.2%) in the third quartile, and 8 (12.3%) in the fourth quartile.

Pleural mesothelioma was the predominant diagnosis, accounting for 56 patients (88.9%) (Table 2). Among the 63 patients with an oncologic diagnosis, 17 (27%) had stage 1 disease, 10 (15.9%) had stage 2 disease, 24 (38.1%) had stage 3 disease, and 11 (17.5%) had stage 4 disease. Stage was not applicable in 1 patient, and 2 patients did not have an oncologic diagnosis. Seven patients (11.1%) received chemotherapy or radiation treatment.

### 3.2. Perioperative Outcomes

The mean hospital length of stay following the index operation was 13.2 ± 9.3 days. Atrial fibrillation was the most observed complication, occurring in 29 patients (44.6%). Pneumonia occurred in 9 patients (13.8%), myocardial infarction in 6 (9.2%), and prolonged mechanical ventilation exceeding 48 h in 3 (4.6%). Myocardial infarction (MI) was ascertained using structured ICD-9 and ICD-10 diagnosis codes for acute and subsequent myocardial infarction documented during index hospitalization. Formal per case clinical adjudication was not performed, i.e., troponin trend, ECG findings, coronary angiography, or a cardiology consult. However, because these codes require clinician-assigned diagnosis, they are expected to primarily capture clinically recognized MI.

Thirteen patients (20%) experienced a readmission, while 20 (30.8%) returned to the emergency department. Returns to the emergency department were most commonly related to management of closed-suction Jacson–Pratt drains, which were left in place after myocutaneous flap reconstruction and associated pain. The single return to the OR was for evacuation of subcutaneous flap hematoma and was managed by the plastic surgery team. Wound infection occurred in 1 patient (1.5%). Mesh infection, pleural space infection or empyema, mesh exposure, chronic seroma, and fistula were tracked as outcomes distinct from wound infection. None of these events were identified in our cohort.

No diaphragmatic reconstruction recurrence was observed during the study period, and no patient required diaphragmatic mesh replacement. A summary of postoperative outcomes is provided in Table 3.

During follow-up, 40 patients (61.5%) died, while 25 (38.5%) were alive at the end of available follow-up. Twenty-seven patients (41.5%) died within the first year following the index operation, 11 (16.9%) died between 1 and 3 years, 1 (1.5%) died between 3 and 5 years, and 1 (1.5%) died between 5 and 8 years. Kaplan–Meier estimated overall survival at 1 year was 58.1 (95% CI, 47.8–67.0%), with 55 patients remaining at risk. Estimated survival at 3 years was 39.3% (95% CI, 29.0–49.4%), with 25 patients at risk, and estimated survival at 5 years was 32.2% (95% CI, 22.0–42.9%), with 14 patients at risk (Figure 1).

### 3.3. Regression Analysis

No statistically significant associations were identified between the evaluated demographic or clinical factors and postoperative outcomes. In the multivariable analysis, cancer stage, chemotherapy or radiation treatment, age, sex, race/ethnicity, and neighborhood deprivation index were not significantly associated with the evaluated outcome (all *p* > 0.05). The adjusted odds ratios were 0.84 (95% CI, 0.14–5.02; *p* = 0.8443) for stage 2–4 disease, 0.54 (95% CI, 0.03–8.46; *p* = 0.6589) for chemotherapy or radiation therapy, 1.11 (95% CI, 0.98–1.26; *p* = 0.0914) for age, 1.02 (95% CI, 0.14–7.27%; *p* = 0.9846) for male sex, 8.15 (95% CI, 0.94–70.29; *p* = 0.0564) for the combined Asian, Black, Hispanic, Native Hawaiian/Pacific Islander, or other race/ethnicity category, and 0.17 (95% CI, 0.02–1.40; *p* = 0.0996) for NDI quartiles 3–4 compared with quartiles 1–2.

Regression modeling was not performed for outcomes with zero or one observed event because of the insufficient number of events for model estimation.

## 4. Discussion

In this 10-year retrospective cohort study, we evaluated clinical outcomes following diaphragmatic reconstruction with Strattice™ biologic mesh in 65 patients undergoing complex thoracic oncologic resection. The cohort was predominantly composed of patients with pleural mesothelioma and was characterized by advanced age and substantial comorbidities. The most notable finding was the absence of diaphragmatic reconstruction recurrence or need for mesh replacement during available follow-up. In addition, postoperative wound infections were rare, occurring only in 1 patient (1.5%). These results contribute valuable evidence to the limited body of literature on biologic mesh in diaphragm reconstruction and suggest that Strattice™ may serve as a viable alternative to traditional synthetic mesh in appropriately selected patients.

Reconstruction of large diaphragmatic defects following oncologic resection poses significant technical challenges. Large and full thickness defects are not amenable to tension-free primary repair and almost always require reconstruction with mesh. Historically, synthetic materials such as PTFE and GoreTex have been the mainstay for diaphragm reconstruction due to their mechanical strength and low elasticity. However, infection rates associated with PTFE vary widely, particularly in contaminated or high-risk surgical fields. For example, in a comparative series of chest-wall and diaphragmatic reconstruction, surgical site complications occurred in 37.5% of patients reconstructed with synthetic mesh compared with 11.5% in patients reconstructed with a bovine biologic mesh, with a readmission rate of 17.5% vs. 0%, respectively [7]. Likewise, late complications such as erosion, rigidity related discomfort, and rare mesh detachment can impair respiratory mechanics and predispose patients to herniation [8].

Biologic meshes are an alternative approach to reconstruction. They have demonstrated superior infection resistance, enhanced host tissue integration, and reduced reoperation rates in various soft tissue reconstructions. Strattice™, specifically, is known for robust revascularization and incorporation, making it an attractive option in contaminated fields or scenarios requiring long-term mechanical durability [9]. At our institution, the preference for biologic over synthetic mesh in diaphragmatic reflects surgeon experience rather than a formal selection protocol. Synthetic mesh infection is perceived as poorly tolerated and, when it occurs, it requires complex explanation which, in patients with oncologic disease, can carry significant morbidity. While widely adopted in abdominal and chest-wall reconstruction, its application in diaphragm reconstruction remains underreported. Our findings address this gap by showing no mesh infections, explantations, or reconstruction site recurrences, during available 10-year follow-up. Given the high-risk nature of mesothelioma surgery and the diaphragm’s anatomical proximity to thoracoabdominal microbial sources, these results underscore the resilience of biologic mesh in a demanding surgical environment.

Overall postoperative morbidity reflects the complexity of thoracic oncologic resection. Postoperative atrial fibrillation was the most frequent complication, occurring in nearly half of the cohort (44.6% of the patients). Postoperative arrythmias are well recognized following major thoracic surgery, particularly after extensive pulmonary and pleural resections [10]. The high rate observed in our cohort likely reflects the magnitude of operation and patient population, although the present analysis was not designed to determine its cause. Prior large institutional series report postoperative pneumonia rates ranging from 10% to 25% [11]. In comparison, our cohort experienced pneumonia in 13.8% of cases and a notably low wound infection rate. These considerations are particularly important for thoracic oncology patients, as their often-multimodal treatment regimen can be impacted by any surgery-related complication. Pneumonia occurred in 13.8% of patients, myocardial infarction in 9.2%, and prolonged mechanical ventilation exceeding 48 h in 4.6%. In addition, 20% of patients experienced readmission, and 30.8% returned to the emergency department. Only one patient returned to the operating room, and one patient experienced stroke. These events should be considered within the context of an older population and a high-risk thoracic oncologic resection and should not be attributed to the reconstructive material. Since this study did not include patients undergoing reconstructive operations with alternative prosthesis, we cannot determine the independent contribution of Strattice™ to overall perioperative morbidity.

Long-term outcomes were driven primarily by the underlying malignant disease. Kaplan–Meier estimated overall survival was 58.1% at 1 year, 39.3% at 3 years, and 32.2% at 5 years. Given that most of our patients had pleural mesothelioma, the survival findings should be interpreted within the context of the underlying disease rather than the reconstructive technique. Published outcomes after surgical management of pleural mesothelioma vary according to stage of the disease, histology, and multimodality treatment [12]. Our study cannot establish whether the choice of reconstructive prosthesis affects survival; rather, the survival analysis provides important information regarding the duration period, over which patients were exposed to potential reconstruction-related complications.

Multivariable analysis did not identify statistically significant associations between the evaluated demographic and clinical characteristics and postoperative outcomes, including pneumonia, wound infection, or readmission, suggesting postoperative outcomes were not disproportionately influenced by demographic or disease factors. This observation is consistent with the potential infection-risk-related benefit of biologic reconstruction in a high-risk population. For thoracic surgeons, Strattice™ offers a combination of structural support and biologic compatibility. Given that our study does not have a comparison group with synthetic mesh, we cannot determine if biologic mesh surpasses synthetic mesh in complex clinical scenarios. Clinically, these findings suggest that biologic mesh may offer a favorable infection profile, as evidenced by the low infection rate. However, without a synthetic mesh comparator, we cannot conclude equivalent or superior durability relative to PTFE. For centers managing mesothelioma or extensive thoracic malignancies, biologic mesh may represent a reasonable reconstructive option. Comparative studies are needed to determine whether it reduces complications associated with synthetic prostheses.

Several limitations should be considered. This was a retrospective cohort study from a single high-volume thoracic center and is subject to potential selection bias and incomplete capture of events occurring outside our healthcare system. The cohort’s demographic composition, reflecting the population served by our center, may also limit generalizability to more diverse patient populations. Furthermore, all reconstructions employed biologic mesh without a synthetic control group, precluding direct comparative risk assessment. The study, therefore, cannot establish comparative superiority of biologic mesh. Granular technical parameters of the reconstruction including mesh dimensions relative to defect size, degree of overlap, and case by case variations in suture counts were not uniformly documented in the operative report for all patients in this retrospective cohort and could not be reported quantitatively. This limits assessment of technique reproducibility and its relationship to outcomes. Our cohort was also relatively small, particularly for uncommon outcomes, limiting statistical power and the precision of multivariable estimates. Several outcomes occurred in zero or one patient, precluding meaningful regression modeling. Finally, because overall survival was primarily influenced by advanced malignancy, death may limit the opportunity to evaluate late prosthesis-related complications.

Despite these limitations, this study provides a decade of institutional experience with Strattice™ mesh in diaphragmatic reconstruction. With a total of 65 patients, this represents the largest reported series of Strattice™-reinforced diaphragm reconstructions to date. The combination of a low rate of wound infection and the absence of diaphragmatic reconstruction recurrence and mesh replacement during available follow-up provides supportive evidence for the durability of biologic reconstructive strategy. Future multicenter studies with larger cohorts, longer follow-ups, and direct comparisons between biologic and synthetic mesh reconstructions are necessary to determine whether the biologic mesh offers significant advantages in infection, durability, and other patient-centered outcomes.

## Figures and Tables

**Figure 1:**
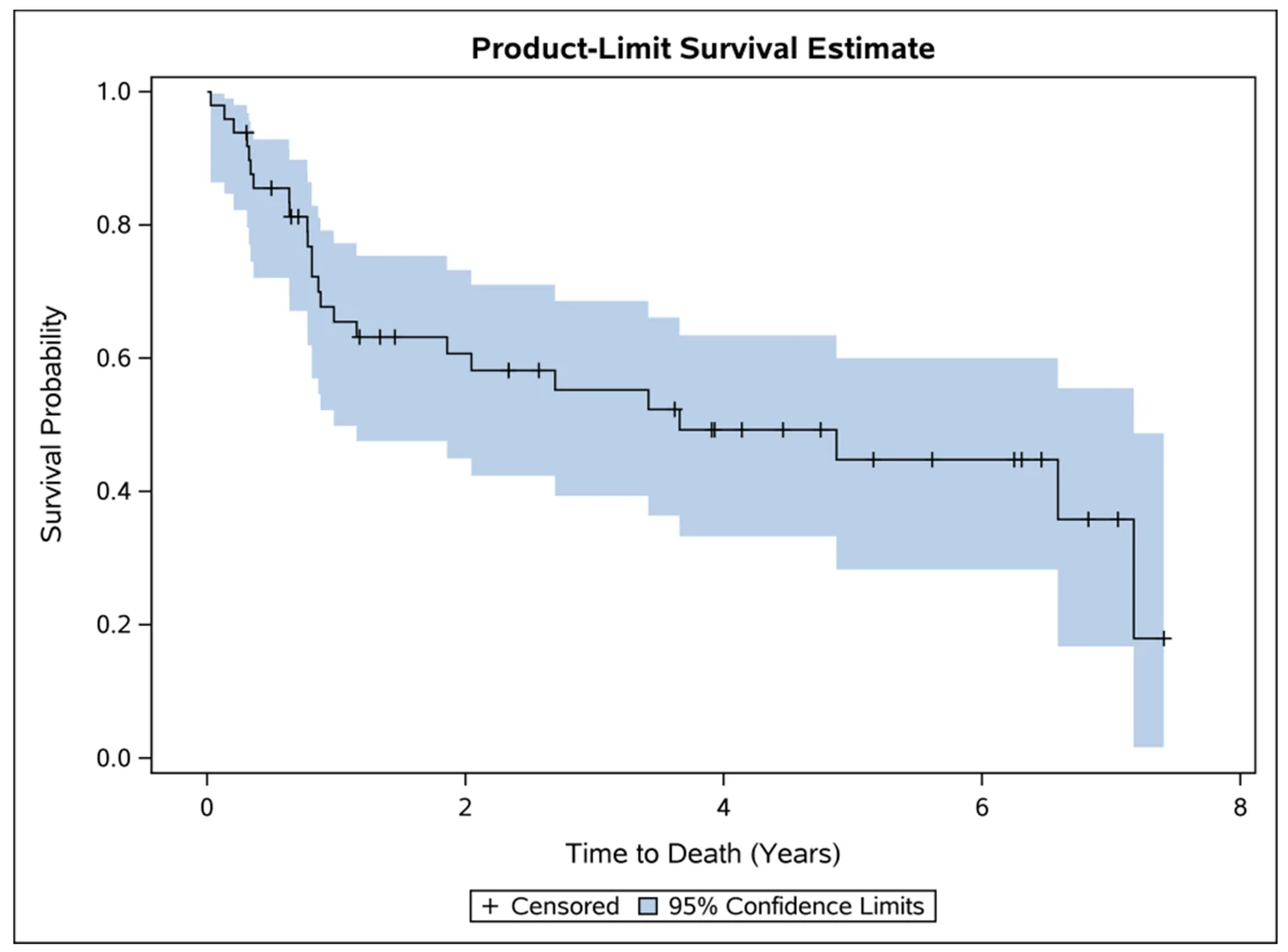
Kaplan–Meier estimate of overall survival following diaphragmatic reconstruction with biologic Strattice™ mesh. The shaded area represents 95% confidence interval, and plus marks indicate censored observations.

**Table 1:** Baseline demographic and clinical characteristics.

Characteristic	Value (*n* = 65)
Age at index operation, mean (SD), years	71.3 (11.6)
Sex, *n* (%)	
Male	42 (64.6)
Female	23 (35.4)
Race and ethnicity, *n* (%)	
White	37 (56.9)
Hispanic	16 (24.6)
Asian	7 (10.8)
Black	4 (6.2)
Native Hawaiian/Pacific Islander or other	1 (1.5)
Insurance status, *n* (%)	
Public	45 (69.2)
Other	20 (30.8)
Neighborhood Deprivation Index quartile, *n* (%)	
1 (lowest)	21 (32.3)
2	19 (29.2)
3	17 (26.2)
4 (highest)	8 (12.3)
Smoking status, *n* (%)	
Never smoker	33 (50.8)
Former smoker	32 (49.2)
Current smoker	0 (0.0)
BMI, mean (SD), kg/m^2^	26.9 (4.7)
BMI, median (IQR), kg/m^2^	27.0 (24.0–29.0)
Elixhauser comorbidity score, excluding cancer, mean (SD)	4.9 (2.4)
Elixhauser comorbidity score, including cancer, mean (SD)	6.3 (2.5)

**Table 2:** Tumor location and histology (*n* = 63).

Tumor Histology/Location (*n* = 63; Excludes 2 Patients Without a Malignant Diagnosis)	*n* (%)
Primary pleural (mesothelioma)	56 (88.9)
Primary cardiac, with chest-wall invasion	2 (3.2)
Primary mediastinal	2 (3.2)
Abdominal malignancy, with diaphragmatic invasion	1 (1.6)
Primary breast, with chest-wall invasion	1 (1.6)
Primary pulmonary	1 (1.6)

**Table 3:** Postoperative outcomes (*n* = 65).

Outcome/Measure, *n* = 65	Value
Hospital length of stay, mean (SD), days	13.2 (9.3)
Follow-up time, mean (SD), years	1.6 (1.9)
Atrial fibrillation, *n* (%)	29 (44.6)
Myocardial infarction, *n* (%)	6 (9.2)
Pneumonia, *n* (%)	9 (13.8)
Prolonged mechanical ventilation (>48 h), *n* (%)	3 (4.6)
Readmission (any inpatient), *n* (%)	13 (20.0)
Return to emergency department, *n* (%)	20 (30.8)
Wound infection, *n* (%)	1 (1.5)
Return to operating room, *n* (%)	1 (1.5)
Stroke, *n* (%)	1 (1.5)
Diaphragm reconstruction recurrence, *n* (%)	0 (0.0)
Diaphragm resection recurrence, *n* (%)	0 (0.0)
Diaphragm mesh replacement, *n* (%)	0 (0.0)
Chest-wall reconstruction recurrence, *n* (%)	0 (0.0)
Chest-wall mesh replacement, *n* (%)	0 (0.0)

SD, standard deviation. Data are presented as mean (SD) for continuous variables and n (%) for categorical variables.

## Data Availability

The data that supports the findings of this study are not publicly available due to privacy and ethical restrictions.
